# Induction of tetrasomy by human papillomavirus type 16 E7 protein is independent of pRb binding and disruption of differentiation

**DOI:** 10.1038/sj.bjc.6601827

**Published:** 2004-04-20

**Authors:** S A Southern, M H Lewis, C S Herrington

**Affiliations:** 1 Department of Pathology, University of Liverpool, Duncan Building, Daulby Street, Liverpool L69 3GA, UK; 2 Bute Medical School, University of St Andrews, St Andrews KY16 9TS, UK

**Keywords:** human papillomavirus, keratinocyte, tetrasomy

## Abstract

We have demonstrated previously that high-risk human papillomaviruses (HPVs) induce tetrasomy in low-grade squamous intraepithelial lesions of the cervix. In this study we show that the E6 and E7 genes of high-risk HPV-16, but not those of low-risk HPV-6, are independently able to induce tetrasomy when constitutively expressed in proliferating monolayer cultures of primary human keratinocytes. Of seven HPV-16 E7 mutants analysed (H2P, Δ6–10, Δ21–24, C24G, S31G/S32G, A50S and S71I), five were severely impaired in their ability to induce tetrasomy in monolayer and raft culture. Only mutant C24G induced tetrasomy to levels comparable with wild-type E7 in monolayer and raft culture. This mutant shows strongly reduced binding to the retinoblastoma gene product pRb. The casein kinase II phosphorylation defective mutant S31G/S32G induced tetrasomy to levels comparable with wild-type E7 in raft culture, but not in monolayer culture, and induction of tetrasomy did not correlate with raft morphology. These results indicate that pRb protein binding is not required for HPV-16 E7 associated tetrasomy and that tetrasomy is not directly related to the ability of this protein to disrupt keratinocyte differentiation.

Human Papillomaviruses (HPV) are small double-stranded DNA viruses with a strict tropism for epithelial cells and 99.7% of cervical cancers are associated with HPV infection (Walboomers *et al*, 1999). Particular HPV types, most notably HPV 16 and 18, predominate in this association and are thus referred to as high-risk types. In previous studies we have shown that basal cell tetrasomy occurs in low-grade squamous intraepithelial lesions of the cervix (SILs) infected with high-risk HPVs but not in those infected with low-risk HPVs (Southern *et al*, 1997). Tetrasomic cells contain four separate copies of each chromosome instead of two. In normal cells, replicated chromosomes remain as tightly associated sister chromatid pairs until mitosis. Sister chromatid separation is stringently regulated and only occurs after each replicated chromosome has become aligned on the mitotic spindle. In tetrasomic cells it would seem that the sister chromatids have become separated but that a subsequent step in mitosis has arrested or failed. Such failures in mitosis can lead to genetic instability and are of cardinal importance in the cancer process.

HPVs encode two small proteins, E6 and E7 that act as potent cell-cycle dysregulators and are able to drive infected cells into S-phase to allow replication of viral DNA. In a productive HPV infection the viral genome is maintained episomally and E6 and E7 are expressed at low levels under viral *cis*- and *trans*-acting control. During neoplastic progression the expression of E6 and E7 is de-repressed, usually as a result of integration of the viral DNA into the host genome. Expression of high-risk E6 and E7 proteins can immortalise primary human epithelial cells *in vitro* (Hawley-Nelson *et al*, 1989) and both proteins bind to numerous cellular target proteins (Kuhne and Banks, 1999; Zwerschke and Jansen-Durr, 2000), several of which are also targets of adenovirus and SV40 early proteins.

The best studied interaction of E7 is that with pRb (Helt and Galloway, 2003). *In vitro* transformation assays of mutant and chimeric E7 proteins from the archetypal high-risk type 16 and the low-risk type 6 have shown that pRb binding affinity correlates with transforming capacity (Heck *et al*, 1992). However, pRb-binding is not sufficient for *in vitro* transformation by HPV-16 E7 (Banks *et al*, 1990). Studies on the interaction of E7 with pRb have yielded the following model. In healthy cells, hypo-phosphorylated pRb associates with the transcription factor E2F and prevents its activation of transcription of many genes required for S-phase progression (e.g. DNA polymerase *α*). As the cell progresses through G1 pRb is sequentially phosphorylated at various sites by cyclin-dependent kinases causing it to release E2F and permit transcription of S-phase genes. Thus pRb, in its hypo-phosphorylated state, can act as a brake on cell-cycle progression and it must be phosphorylated to allow the cell-cycle to continue to S-phase. HPV-16 E7 protein binds to hypo-phosphorylated pRb preventing its sequestration of E2F and potentiating its proteolytic degradation. This effectively removes one of the main nodes of cell-cycle regulation at this point and facilitates unscheduled progression into S-phase. This is potentiated by the direct binding of E7 to E2F1 with consequent activation of E2F1-drive transcription (Hwang *et al*, 2002). The role of pRb is not limited to the G1/S boundary of the cell cycle. pRb deficient fibroblasts lose the ability to sustain a G2 arrest in the presence of mitotic blocks and proceed on into G1 and then S-phase without an intervening cell division (Di Leonardo *et al*, 1997; Niculescu *et al*, 1998). Furthermore, pRb functionally interacts, specifically during G2/M, with Hec1p, a protein required for proper chromosome segregation (Zheng *et al*, 2000). The transcription factor E2F is also involved at G2/M. DNA microarray analysis has shown that those genes transcriptionally regulated by E2F fall into two classes; those expressed at G1/S and another class expressed at G2/M (Ishida *et al*, 2001).

pRb is one member of a family of proteins that share a ‘pocket’ domain involved in protein/protein interactions. The three members of the pocket-protein family, pRb, p107 and p130, seem to have largely overlapping functions (Mulligan and Jacks, 1998). While pRb is mutated or otherwise inactivated (e.g. by HPV E7) in most human cancers, loss of function of p107 or p130 is rarely, if ever, observed. p130 predominates in quiescent or terminally differentiated cells and p107 predominates in proliferating cells. Although E7 binds to all three proteins via the pocket domain it preferentially binds p130 (Smith-McCune *et al*, 1999).

In a previous study we showed that tetrasomy occurs in organotypic raft culture of primary human keratinocytes (PHKs) expressing the (high-risk) HPV-18 E7 gene alone under control of the native, differentiation dependent, HPV-18 enhancer/promoter (Southern *et al*, 2001). Expression of HPV-18 E6 protein alone under these conditions did not cause tetrasomy. However, the E6 and E7 proteins both disrupt G2/M checkpoints *in vitro* (Thomas and Laimins, 1998). Hence, in the present study, we test the hypothesis that the E6 and E7 proteins of high-risk HPV can induce tetrasomy when expressed constitutively. We also test the hypothesis that the induction of tetrasomy by E7 is a result of the E7 protein's interference with pRb function.

## MATERIALS AND METHODS

### Cell lines and culture

Primary human keratinocytes from neonatal foreskin (Clonetics) were cultured in keratinocyte growth medium 2 (Clonetics). Retroviral packaging cell lines were obtained from the American Type Culture Collection (PA317 containing wild-type HPV genes; Halbert *et al*, 1992) or given by Dr Denise Galloway (PG13 containing mutant HPV-16 E7 genes; Demers *et al*, 1996) and were cultured in Dulbecco's modified Eagle's medium with 10% foetal calf serum.

### Transduction and selection of PHKs

Fresh medium was applied to confluent cultures of packaging cells and allowed to accumulate virus overnight. The medium was filtered through 0.45 *μ*m cellulose acetate filters and combined with an equal volume of keratinocyte growth medium 2 containing hexadimethrine bromide (polybrene) (Sigma H9268) to a final concentration of 6 *μ*g ml^−1^. In total, 4 ml of this mixture was applied to passage 1 monolayer PHKs at approximately 10% confluency in 25 cm^2^ flasks. After 6 h the medium was removed, the cells were rinsed in PBS and fresh medium was added. Selection was begun 24 h later with G418 at 150 *μ*g ml^−1^ and continued for 48 h. Mock-infected cultures treated this way were dead by day 3 after selection.

### Organotypic raft culture

A detailed protocol is described elsewhere (Wilson *et al*, 1992). Briefly, 10^6^ keratinocytes were seeded onto a collagen plug containing 5 × 10^5^ J2-3T3 feeder cells in one well of a six-well plate and submerged in raft culture medium. When confluent the plug was transferred onto a wire mesh grid in a 10 cm Petri dish to raise it to the air/liquid interface above raft culture medium. The cultures were grown for 10 days then fixed in 10% neutral buffered formalin for 2 days and embedded in paraffin wax. Raft culture medium is 70% Dulbecco's modified Eagle's medium, 25% Ham's F12 medium, 5% foetal calf serum supplemented with 180 *μ*M adenine, 5 *μ*g ml^−1^ insulin, 400 ng ml^−1^ hydrocortisone, 5 *μ*g ml^−1^ transferrin, 200 pM 3,3′,5-triiodo-_L_-thyronine (Sigma T6397), 100 pM cholera toxin, 5 ng ml^−1^ epidermal growth factor, 2.5 *μ*g ml^−1^ fungizone (Life Technologies), 100 U ml^−1^ penicillin G and 100 *μ*g ml^−1^ streptomycin.

### PCR rescue and sequencing

Genomic DNA was prepared using a Qiagen blood/tissue-culture small scale DNA isolation kit. LXSN vector specific flanking primers were used in standard PCR conditions to amplify an approximately 400 bp product, which was gel-purified and subjected to automated sequencing of one strand. The results were unambiguous in each case.

### Interphase cytogenetics

Monolayer cells were trypsinized, allowed to attach to glass slides overnight in a droplet of medium in a CO_2_ incubator at 37°C and fixed by immersion in 10% neutral buffered formalin for 2 days. Slides were previously treated with aminopropyltriethoxysilane (Sigma A3648). 6 *μ*m sections of paraffin embedded raft cultures were taken onto treated slides, heated overnight at 60°C, dewaxed in xylene and rehydrated through an ethanol series. The method of hybridisation is described in detail elsewhere (Southern and Herrington, 1996). Briefly, slides were pretreated with sodium thiocyanate, pepsin/HCl and hybridized with biotin or digoxigenin labelled alpha-satellite probes specific to either chromosome 1, 3 or 17. The chromosome 1 probe was kindly provided by Dr AH Hopman (University of Limburg, Maastricht, the Netherlands) and the chromosome 3 and 17 probes were purchased from Appligene/Oncor. Signal was detected with peroxidase conjugated antibodies, diaminobenzidine and hydrogen peroxide.

### Scoring tetrasomy

**Figure 1:**
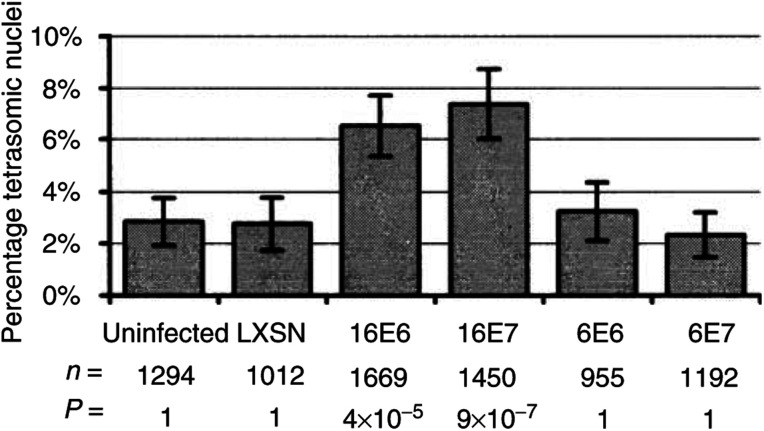
Percentage tetrasomy induced in monolayer culture of PHKs expressing of HPV-16 and HPV-6 E6 and E7 proteins. Indicated beneath each bar is the number of nuclei counted (*n*) and the Bonferroni corrected *P*-value (*P*) based on a *χ*^2^ test using two-way contingency tables compared to uninfected. The error bars show the 95% confidence interval assuming a normal distribution.

Nuclei were classified at × 630 magnification into five classes depending on the number of spots present: 1, 2, 3, 4 and 5+ spots. In monolayer culture the percentage tetrasomy is the number of 4-spot nuclei divided by the total × 100. In raft culture sections many nuclei are truncated. We have shown previously that sectioned material from a purely tetrasomic population produces a signal distribution containing approximately equal numbers of nuclei containing 3 and 4 spots (Southern and Herrington, 1996). Therefore, the 3- and 4-spot classes were combined and classed as tetrasomic. The *P*-values derived using *χ*^2^ comparisons were multiplied by the number of comparisons, 6 in Figure 1

and 8 in Figure 2

**Figure 2:**
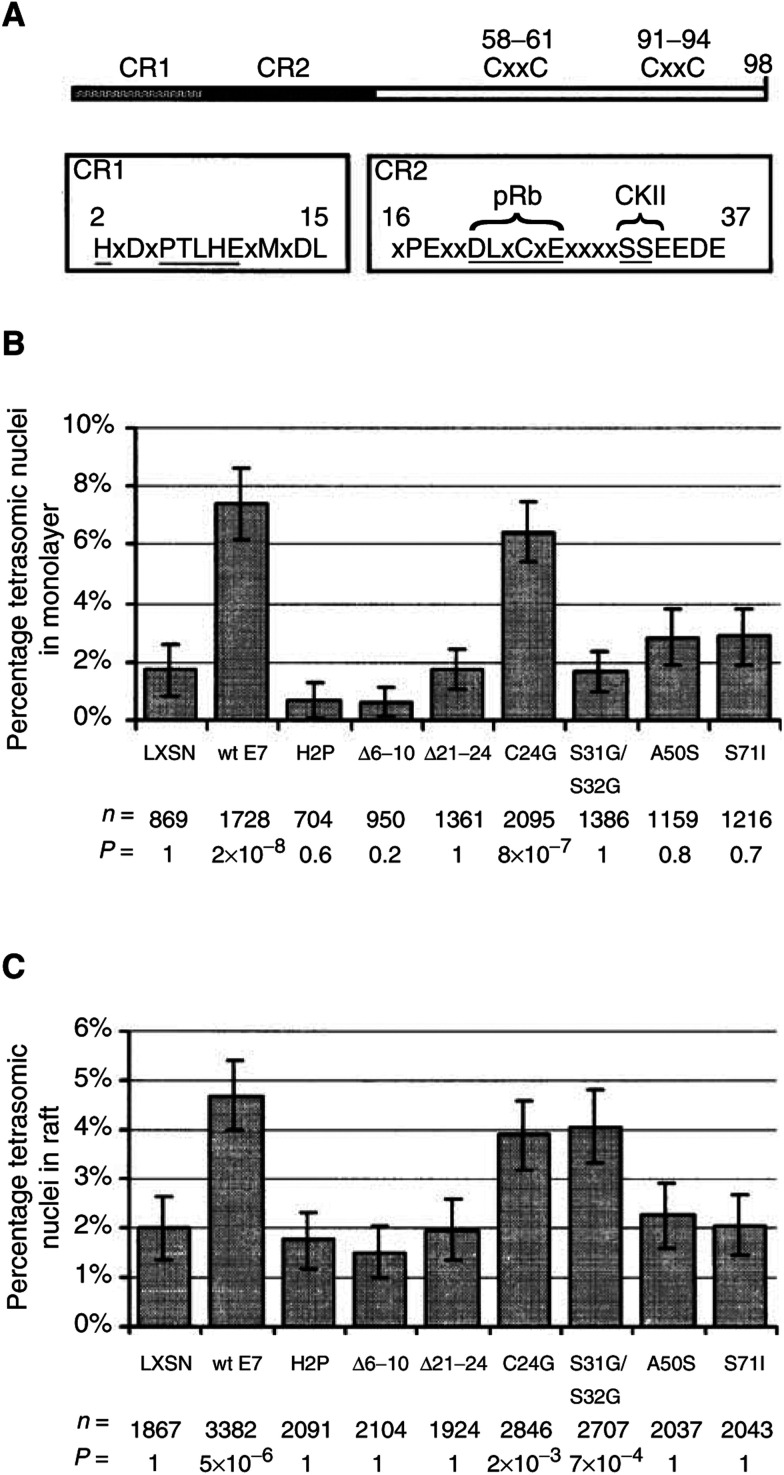
Induction of tetrasomy by HPV-16 E7 mutant proteins. (**A**) Schematic diagram of HPV-16 E7 protein showing sequence homology with adenovirus E1a in the conserved regions CR1 and CR2. Positions of mutations in CR1 and CR2 are underlined. The locations of pRb binding and CKII phosphorylation sites are indicated. (**B**) Percentage tetrasomy induced by mutant HPV-16 E7 proteins in monolayer culture of PHKs. (**C**) Percentage tetrasomy induced by mutant HPV-16 E7 proteins in organotypic raft culture of PHKs. Indicated beneath each bar is the number of nuclei counted (*n*) and the Bonferroni corrected *P*-value (*P*) based on a *χ*^2^ test using two-way contigency tables compared to LXSN. The error bars show the 95% confidence interval assuming a normal distribution.

(a Bonferroni correction), to avoid type I errors (Altman, 1991). 95% confidence interval is −1.96SE to +1.96SE.

## RESULTS

### The E6 and E7 genes of HPV type 16, but not those of type 6, are independently able to induce tetrasomy in primary human keratinocyte monolayer culture

We investigated constitutive expression of both the E6 and E7 genes of the archetypal high-risk type 16, and also of the low-risk type 6 HPV, by obtaining PA317 retrovirus packaging cell lines generated in the laboratory of Dr Denise Galloway (Halbert *et al*, 1992), which contain the following genes inserted into the LXSN retroviral vector: HPV16 E6 & E7, HPV6 E6 & E7 and LXSN vector alone (ATCC, Manassas, VA, USA). In these constructs the inserted genes are transcribed from the constitutive promoter in the moloney murine leukaemia retroviral LTR. Primary human keratinocytes growing as submerged monolayers were transduced with retrovirus at passage 1, selected with G418 for 2 days and then allowed to expand. The cultures were fixed and analysed at passage 3 (7 days after transduction for the HPV-16 constructs, 10–14 days for the others). The cells were analysed by interphase cytogenetics using biotinylated DNA probes specific for chromosomes 1, 3 and 17. We have previously shown that HPV-induced tetrasomy involves at least chromosomes 1, 3, 4, 6, 10, 11, 17, 18 and X (Giannoudis *et al*, 2000). The results are shown in Figure 1. Significantly increased levels of tetrasomy occured in cultures transduced with retroviruses expressing HPV16 E6 and E7 genes. Those cultures transduced with retroviruses expressing HPV6 E6 or E7, as well as cultures transduced with LXSN vector, showed no significant increase in levels of tetrasomy above uninfected PHKs. Thus, the ability of these HPV proteins to induce tetrasomy correlates with their ability to transform cells *in vitro*.

### High affinity pRb binding is not required for induction of tetrasomy by HPV16 E7

In order to map the domains of E7 required for its induction of tetrasomy, we investigated seven HPV16 E7 mutants: H2P, Δ6–10, Δ21–24, C24G, S31G/S32G, A50S and S71I. These mutants, the gift of Dr Denise Galloway (Demers *et al*, 1996), were in the form of LXSN constructs in PG13 retrovirus packaging cell lines. HPV-16 E7 protein has been notionally divided into domains based on sequence homology to widely separated regions of the much larger adenovirus E1a protein. Mutations H2P and Δ6–10 are located in conserved region 1 (CR1), mutations Δ21–24, C24G and S31G/S32G are located in CR2 and mutations A50S and S71I are located close to the C-terminal metal-binding domain (Figure 2A). PHKs were transduced at passage 1, selected for 2 days, expanded for 3–4 days then split and grown in parallel as submerged monolayer and organotypic raft cultures. Expression of the mutant proteins under these conditions has been demonstrated previously (Demers *et al*, 1996). Monolayer cultures were fixed at passage 3. Raft cultures were grown for 10 days before fixation. After fixation the presence of the correct mutant gene was confirmed in each case by PCR rescue and sequencing. Tetrasomy was scored by interphase cytogenetics using biotinylated DNA probes specific for chromosomes 1, 3 and 17. Only the C24G mutant E7 protein was able to induce levels of tetrasomy similar to wild-type HPV16 E7 in monolayer culture (Figure 2B). The other mutants all showed strongly reduced levels of tetrasomy with least loss of effect being shown by the C-terminal mutants A50S and S71I. In raft culture the same pattern emerged with the exception that the S31G/S32G protein gave a similar level of tetrasomy to wild type and C24G (Figure 2C). The tetrasomy was predominantly suprabasal (data not shown).

### The ability of HPV16 E7 to induce tetrasomy is separable from its ability to disrupt keratinocyte differentiation in organotypic raft culture

**Figure 3:**
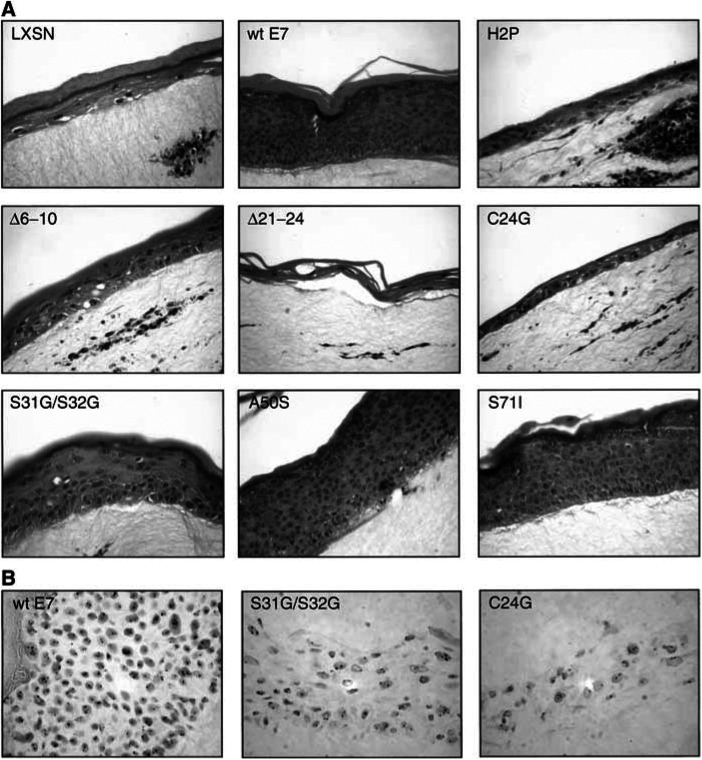
(**A**) Haematoxylin and eosin stained sections of organotypic raft cultures of PHKs transduced with retrovirus expressing HPV-16 E7 mutant proteins. Magnification × 400. (**B**) Interphase cytogenetic staining of a raft culture section from each of the three morphological categories with a chromosome 17 specific probe. Nuclei counterstained with haematoxylin. Magnification × 630.

Haematoxylin and eosin stained sections show three different morphological groups in the organotypic raft cultures (Figure 3

). In the first group, represented by wild-type HPV-16 E7 and the mutant proteins A50S and S71I, a thick epithelium is formed containing uniformly undifferentiated cells throughout. The second group, represented by the mutant proteins H2P, Δ6–10, Δ21–24 and C24G, as well as the LXSN vector, forms a very thin epithelium containing differentiated cells. The third group, represented only by mutant protein S31G/S32G, shows a thick epithelium with distinct layers of basal (undifferentiated) and spinous (differentiated) cells. Those E7 proteins inducing tetrasomy in raft culture do not fall into any particular morphological class. Induction of tetrasomy occurs in rafts from each class, namely HPV-16 E7 wild type, C24G and S31G/S32G. Thus, the ability of HPV-16 E7 mutant proteins to induce tetrasomy does not correlate with any particular effect on keratinocyte differentiation in raft culture.

## DISCUSSION

In earlier studies we have shown that tetrasomy occurs in a proportion of low-grade SILs infected with high-risk HPV types but does not occur in SILs infected with low-risk HPV types (Giannoudis *et al*, 2000). We also identified tetrasomy in suprabasal keratinocytes in raft cultures expressing HPV 18 E7, but not in rafts expressing E6, under transcriptional control of the native HPV-18 enhancer/promoter (Southern *et al*, 2001). However, the HPV-18 enhancer/promoter is strongly repressed in basal cells and becomes derepressed when the cells migrate up the epithelium and begin to differentiate. Hence, the ability of these genes to induce tetrasomy in proliferating cells cannot be assessed. Moreover, both E6 and E7 have been shown independently to disrupt G2/M cell-cycle checkpoints *in vitro* (Thomas and Laimins, 1998). Therefore, in this study, we tested the hypothesis that constitutive expression of both of these genes can induce tetrasomy in proliferating cells. In monolayer culture, which most closely approximates the basal/parabasal proliferating layer of an epithelium, both E6 and E7 of HPV-16 were able to induce tetrasomy. In contrast, the E6 and E7 genes of the low-risk HPV type 6 were not able to induce tetrasomy. These findings are consistent with the known biochemical properties of these proteins.

To investigate further the induction of tetrasomy by HPV-16 E7 protein, we analysed monolayer and raft cultures of PHKs transduced with retroviruses expressing mutant E7 proteins. These seven are distributed throughout the protein and are well characterised with respect to pRb-binding and transformation (Demers *et al*, 1996). All but one of the mutants showed a significant reduction in tetrasomy compared to wild-type E7 in monolayer culture. This suggests that no single domain of the E7 protein is responsible for the effect and that induction of tetrasomy is an indirect downstream effect of two or more E7 functions residing in different parts of the protein.

The one mutant protein that did not cause a reduction in tetrasomy was C24G. This substitution to glycine of the central cysteine of the xLxCxE motif has been reported to abrogate binding to pRb (Davies *et al*, 1993; Demers *et al*, 1996) and p130 (Smith-McCune *et al*, 1999), and to either reduce (Davies *et al*, 1993) or abrogate (Demers *et al*, 1996) binding to p107. This suggests that pRb-binding is not required for induction of tetrasomy. The related mutant Δ21–24, a deletion of xLxC from the xLxCxE motif, is also unable to bind pRb or p107 (Davies *et al*, 1993; Phelps *et al*, 1992) but shows no induction of tetrasomy. What is the functional difference between the C24G and the Δ21–24 mutations? Both mutant proteins are unable to disrupt keratinocyte differentiation (Demers *et al*, 1996 and see Figure 3A), transactivate the adenovirus E2 promoter (Edmonds and Vousden, 1989; Phelps *et al*, 1992) or transform cells *in vitro* (Edmonds and Vousden, 1989; Phelps *et al*, 1992). However, the C24G mutant protein is able to stimulate proliferation in immortalized rodent fibroblasts (Caldeira *et al*, 2000). Furthermore, recent quantitative binding studies have shown that the C24G mutant retains 30% of the pRb-binding affinity of the wild-type E7 protein (apparent K_D_s of 14 and 4.5 nM respectively) (Dong *et al*, 2001). Nevertheless, this mutant, which exhibits 70% reduction in pRb binding affinity, was able to induce a similar of level of tetrasomy to wild-type protein. Thus, it can still be inferred that induction of tetrasomy by E7 is not directly related to pRb-binding. However, if C24G retains or accentuates some difference in binding preference between the pocket proteins then the direct involvement of p107 or p130 cannot be excluded. Alternatively, the mechanism of induction of tetrasomy may be independent of pocket-protein binding altogether. The Δ21–24 deletion mutant is completely unable to bind to pRb and does not induce tetrasomy. The difference between the findings for this mutant and those for C24G may in part be related to a change (possibly conformational) that impinges on some other E7 function additional to pocket-protein binding that is not affected by C24G. The other mutants analysed are able to bind to pRb but, with the exception of S31G/S32G in raft culture, are unable to induce tetrasomy. Thus, induction of tetrasomy does not segregate with pRb binding.

In organotypic raft culture C24G was joined by S31G/S32G in possessing the ability to induce tetrasomy, which was predominantly suprabasal, to levels comparable with wild-type protein. The two serines at positions 31 and 32 form part of the recognition site for, and are phosphorylated by, casein kinase II. It is not clear why the S31G/S32G mutant cannot induce tetrasomy in monolayer but is capable of doing so in raft culture, but this differential ability is compatible with our previous hypothesis that the mechanisms of induction of basal and suprabasal tetrasomy are different (Southern *et al*, 2001). In monolayer culture the cells are undifferentiated and cycling and most closely resemble the basal/parabasal layer of an epithelium. Expression of E7 protein in these cells may disrupt cell-cycle checkpoints, leading to tetrasomy, and our data suggest that this property requires phosphorylation of the S31/S32 site. In raft culture the keratinocytes form a squamous epithelium and the E7 protein induces unscheduled DNA synthesis in a proportion of postmitotic, differentiated keratinocytes (Cheng *et al*, 1995). Our data suggest that phosphorylation at the S31/S32 site is not required for this function. Only the S31G/S32G mutant gave a raft morphology indicating both proliferation (a thickened raft) and differentiation. Therefore, mutation of the CKII phosphorylation site reduces the ability of the E7 protein to disrupt keratinocyte differentiation but has no effect on induction of tetrasomy. Further analysis of other mutations in the CR2 region of HPV-16 E7 may clarify the mechanism.

The other rafts were either proliferative but relatively undifferentiated (wild-type, A50S, S71I) or nonproliferative (vector, H2P, Δ6–10, Δ21–24, C24G), in agreement with the findings of Demers *et al* (1996). The induction of tetrasomy by C24G, S31G/S32G and wild-type E7 demonstrates that this property is not related to the ability of the E7 protein to disrupt keratinocyte differentiation. The proportion of keratinocytes with detectable tetrasomy was relatively low but this is compatible both with our previous study (Southern *et al*, 2001) and with observations reported by others (Chien *et al*, 2002). The latter study provided evidence that postmitotic, differentiated keratinocytes expressing high-risk HPV E7 protein have alternative fates, with only a minority undergoing endoreduplication, consistent with our data.

The absence of tetrasomy in rafts expressing C-terminal mutants A50S and S71I is consistent with the observation that, despite their ability to bind to pRb, C-terminal mutations inhibit the ability of E7 to induce S phase and endoreduplication in differentiated keratinocytes and is compatible with the requirement for inactivation of both pRb and p21 by HPV 16 E7 in order to bypass cell-cycle arrest (Helt *et al*, 2002). The requirement for factors additional to abrogation of the p16INK4A/pRb checkpoint for induction of centrosome abnormalities during keratinocyte immortalisation supports the involvement of additional E7 functions (Piboonniyom *et al*, 2003). Thus, centrosome abnormalities were demonstrable in keratinocytes immortalised by a combination of HPV 16 E7 and hTERT but not in those immortalised by cdk4/hTERT or by hTERT alone, the latter having lost expression of p16INK4A.

In summary, both the E6 and E7 proteins of HPV 16 can induce tetrasomy in proliferating keratinocytes. Mutational analysis of the E7 protein shows that the ability of E7 to induce tetrasomy is separable from both pRb binding and disruption of keratinocyte differentiation, indicating that this phenomenon is not directly related to either of these properties and is likely to require multiple functions of the E7 protein.
